# Computational tools to investigate genetic cardiac channelopathies

**DOI:** 10.3389/fphys.2013.00390

**Published:** 2013-12-26

**Authors:** Hugues Abriel, Enno de Lange, Jan P. Kucera, Gildas Loussouarn, Mounir Tarek

**Affiliations:** ^1^Department of Clinical Research, University of BernBern, Switzerland; ^2^Department of Knowledge Engineering, Maastricht UniversityMaastricht, Netherlands; ^3^Department of Physiology, University of BernBern, Switzerland; ^4^INSERM, UMR 1087, l'Institut du thoraxNantes, France; ^5^Centre National de la Recherche Scientifique, L'Institut du Thorax, UMR 6921Nantes, France; ^6^L'Institut du Thorax, UMR 6921, Université de NantesNantes, France; ^7^Theory, Modeling and Simulations, UMR 7565, Université de LorraineVandoeuvre-lés-Nancy, France; ^8^Theory, Modeling and Simulations, UMR 7565, Centre National de la Recherche ScientifiqueVandoeuvre-lés-Nancy, France

**Keywords:** ion channels, channelopathies, computer models, simulation, molecular dynamics

## Abstract

The aim of this perspective article is to share with the community of ion channel scientists our thoughts and expectations regarding the increasing role that computational tools will play in the future of our field. The opinions and comments detailed here are the result of a 3-day long international exploratory workshop that took place in October 2013 and that was supported by the Swiss National Science Foundation.

## Introduction and current situation

Ion channels are essential proteins that are located at the plasma membrane of virtually all cells. They allow for the passage of ions across the plasma membrane according to their electro-chemical gradients (Hille, 2001). Most of these ion channels show selectivity in their permeability properties (i.e., to sodium, potassium, calcium, or chloride ions found in physiologic fluids). Ion channel gating (opening and closing features) plays a key role in the regulation of ion channel activity. Many ion channel proteins are able to sense the electrical field across the cellular membrane, which is negative at a resting state and inverts upon depolarization of the membrane (Armstrong and Hille, 1998; Bezanilla, 2005). Such channels are said to be voltage-gated, since they open (activate) upon depolarization (Loussouarn and Tarek, 2012). Following depolarization-dependent opening, some of these channels will inactivate and prevent the permeation of further ions.

The essential role of ion channels, particularly in the control of cellular excitability and epithelial transport, was demonstrated at the beginning of the 20th century, even before much was known of their molecular nature (Hille, 2001). In the 1950s, it became clear that ion channels are the targets of very potent drugs that are used to treat a broad range of human pathologies, such as pain, cardiac arrhythmias, hypertension, and epilepsy.

Thanks to the major progress in molecular and human genetics in the 1980s, a myriad of genetic mutations were found in the genes coding for different ion channel subunits. This led to the definition of *genetic channelopathies*, which groups together all disorders that are primarily caused by dysfunctional mutant ion channels (Ashcroft, 2006). Channelopathies are known to cause epilepsy, pain syndromes, migraines, periodic paralysis, myotonia, cardiac arrhythmias, hypertension, hypotension, cystic fibrosis, and many other rare genetic disorders (Ashcroft, 2006).

Mutations in ion channel subunit genes have been shown to alter ion channel function in many different ways (Cannon, 2007). Mutations can lead to either a loss-of-function (i.e., resulting from a defect in biosynthesis), or to a gain-of-function (i.e., resulting from the alteration of inactivation properties). Peroz et al. (2008), however, demonstrated that many of the molecular dysfunctions do not abide by this simple binary classification. There are, for example, dysfunctions that affect protein targeting, the binding of partner proteins, and the regulation by phospholipids. The demonstration of the important roles of ion channels in numerous diseases, coupled with the fact that ion channels are excellent drug targets because of their location at the cellular membrane, have stimulated the field of ion channel research over the last three decades (Ranjan et al., 2011). It is estimated that about 10,000–15,000 original scientific articles are published every year on the topic of ion channels. Most of the research activity is focused on ion channel-related pathophysiological mechanisms, as well as on finding new diagnostic, preventive, and therapeutic strategies to treat and/or prevent disease.

A broad community of scientists is working to address specific questions about the function and roles of ion channels in disease. Included in this community are biophysicists, biochemists, geneticists, physiologists, pharmacologists, computational biologists, and clinical scientists. They are all tackling these questions with their specific expertise.

Because of the overwhelming amount of data, attempts to integrate this highly fragmented knowledge from vastly different disciplines have been proven to be difficult. One major objective of summarizing the information is to enable its application in clinical practice. Biophysicists, computational biologists, and biochemists are all playing an important role to achieve this objective. The seminal work of Hodgkin and Huxley (1952) on the giant axon of the squid (Nobel Prize 1963) initiated this approach. Applying the strategy of Hodgkin & Huxley, Noble and his colleagues began developing very soon thereafter, a number of models for various types of cardiac cells and tissues (for a review, see Noble et al., 2012). As a result of the developments in information technology over the last few decades, the modeling and simulation of biophysical and biological processes has consequently become much more amenable. Mathematical models of ion channels and excitable tissues have become increasingly refined and complex, while constantly taking into account new experimental findings.

The current trend in ion channel modeling is to represent ion channels as Markovian systems, which generalizes the framework established by Hodgkin and Huxley (1952). Such models recapitulate single channel behavior, whole cell currents, and the action of drugs (Irvine et al., 1999; Clancy et al., 2007; Milescu et al., 2008; Bett et al., 2011). Many models have been developed at the cellular level, notably cardiac cells [e.g., the review by Wilhelms et al. on human atrial cell models (Wilhelms et al., 2013)]. Notably there is an increasing level of structural detail in cell models. For example, cardiac models have started to incorporate the fine structure of the sarcoplasmic reticulum, the T-tubule system and the dyadic spaces (Williams et al., 2011; Nivala et al., 2012). Simulation, using computational methods, is becoming an integral part of the research process in the biomedical sciences, as illustrated by a recent study by Silva et al. (2009). At this stage, these computational approaches have mainly been used to investigate the possible consequences of the mutation-induced alterations of voltage-gated ion channels on the excitability of cells in neurologic disorders such as myotonia (Cannon, 2007), cardiac arrhythmias, and conduction defects (Rudy, 2012). Simulations of more integrative signals have also been performed, such as the recorded cardiac electrical activity on the body surface (ECG) (Rudy, 2012) and the electrical activity of circuits with hundreds of neurons (Hill et al., 2012). An outstanding example of such a current project is the “Human Brain Project,” which has recently been selected as one of the research flagship projects of the European commission (Markram, 2012).

Another important trend is the emerging and very promising field of research using computational frameworks to model molecular function (molecular dynamics), for which the Nobel Prize of Chemistry was awarded to Karplus, Levitt, and Warshel in 2013. These computational approaches are now feasible for very complex molecules, such as proteins and multiprotein complexes, i.e., ion channels (Gosselin-Badaroudine et al., 2012; Tarek and Delemotte, 2013). These tools will most likely play an increasing role for membrane proteins, as the rate of newly identified structures from crystallography experiments is growing exponentially (http://blanco.biomol.uci.edu/mpstruc/), with 433 unique proteins identified as of November 2013.

## The future of modeling to study ion channels and channelopathies

A model can be defined as a set of rules, ideally based on the laws of physics and expressed with the formalism of mathematical equations, to describe an observed system. The aim of computer simulation is to implement such a model in a computer program with the goal of replicating the behaviors observed in the real world. To be as close as possible to physical reality, one should strive to formulate models based on established physical principles as long as the experimental data allows for this. However, in practice, this is not always possible, and one often recourses to phenomenological models to describe ion channel behavior. Nevertheless, such models are still highly valuable.

Future modeling developments will most likely be performed using multidisciplinary and multiscale approaches that encompass the modeling of molecules (molecular dynamics), the modeling of ion channel microscopic kinetics, the modeling of cellular electrical activity, and finally, the modeling of the electrical properties of tissues and organs. There is a need to bridge the gap between molecular dynamics simulations and kinetic models of ion channels. The derivation of energy landscapes from molecular dynamics data, and thus, the rate constants in Markovian kinetic models, is an excellent approach that was recently pioneered by Rudy's group, (Silva et al., 2009; Nekouzadeh and Rudy, 2011) where effects of mutations in the KCNQ1 -mediated current were accurately predicted, purely based upon protein sequence. Whether or not such modeling should be performed at all levels remains uncertain. Multiscale modeling is a synergetic process, thus, it seems reasonable that all levels should be modeled and integrated to warrant an optimal yield of information.

## Collaboration and multidisciplinarity are essential

There is a tremendously large amount of data available on ion channel biology and function, as well as on channelopathies. There is a critical need to regroup this information into well-constructed databases, which incorporate as much detailed and quality information about ion channels and their generated currents as possible. Collaboration between experimentalists and modeling scientists must be encouraged in order to obtain an optimal yield of information to produce relevant new knowledge. Both scientific individuals, if not united in the same person, and groups should work closely together on projects. Collaborative platforms and interfaces could also encourage a structured exchange of information in both directions. Psychological barriers that may be present on either side could be relieved by working together in the same research group or consortium.

## Current cellular electrophysiology protocols

Currently, most of the electrophysiology protocols that are used to record the activity of ion channels from whole-cell recordings do not have the most optimized design to enable the collection of all the intrinsic dynamics of ion channels. There is a need to generate, refine, and use non-conventional cellular electrophysiology protocols which subject the ion channels to continuous perturbations that increase the information obtained from such experiments. An inspiring example is the “dichotomous noise” protocol proposed by Millonas and Hanck (1998). Compared to models based on the Hodgkin-Huxley formalism, Markov models also bring additional challenges in terms of parameter estimation, identifiability, and computational speed. These challenges call for the development of innovative methods to estimate model parameters, to formulate Markov models that are mathematically identifiable, and to allow automatized identification from experimental data that are obtained with optimized voltage clamp protocols. Several research groups have already started to address these different issues (Bruno et al., 2005; Fink and Noble, 2009; Siekmann et al., 2012). All of these approaches could be aptly named “Systems Biophysics,” and should be further developed. Furthermore, the experimentalists should consider “full ion channel (kinetic) models” when communicating their data. Raw data should also be made available, rather than only a summary of activation and inactivation curves, for example. If this had been done in past publications, it may be postulated that there would not have been a need for the “channelome” project of the Swiss Institute of Technology of Lausanne (Ranjan, 2011).

## “variability” as a parameter to take into account

Experimental results show “variability” in responses following administration of a given stimulus to biological samples. The sources of this variability are diverse and may reflect either intrinsic biological properties or may result from the recording set-up. With few exceptions (Sato et al., 2010; Lemay et al., 2011), the variability and its consequences have not been adequately addressed by the current models which oftentimes calculate averaged values. This problem must be addressed in the future.

## Genetic complexity

It is conceptually simple to model the consequences of mutations in ion channel genes which cause well-defined phenotypes, such as inherited epilepsy or congenital long QT syndrome, that are currently described as monogenic disorders. However, this simple paradigm of “one mutation causing one phenotype” does not hold true in many cases (Probst et al., 2009; Klassen et al., 2011; Bezzina et al., 2013). As a consequence, these observations should be used in future models by incorporating gene-gene and gene-environment interactions.

## Conclusions

In conclusion, we are convinced that modeling, through the use of more and more sophisticated computational tools, will prove to be an essential component for the investigation of channelopathies. In some fields of experimental science such as chemistry, computer modeling is already an essential part of the publication process. In the near future, no one will be able to simply say, “I do not believe in computer models!”

### Conflict of interest statement

The authors declare that the research was conducted in the absence of any commercial or financial relationships that could be construed as a potential conflict of interest.
